# Proteome of Renal Tubuli and Serum Differentiate Pre‐Existing Type 2 Diabetes and Post‐Transplant Diabetes in Kidney Transplant Recipients

**DOI:** 10.1002/prca.70000

**Published:** 2025-02-24

**Authors:** Eleni Skandalou, Mariell Rivedal, Hans‐Peter Marti, Thea A. S. Halden, Trond Jenssen, Bjørn Egil Vikse, Anders Åsberg, Jessica Furriol

**Affiliations:** ^1^ Department of Clinical Medicine University of Bergen Bergen Norway; ^2^ Department of Medicine Haukeland University Hospital Bergen Norway; ^3^ Department of Transplantation Medicine Oslo University Hospital Rikshospitalet and University of Oslo Oslo Norway; ^4^ Metabolic and Renal Research Group Faculty of Health Sciences UiT The Arctic University of Norway Tromsø Norway; ^5^ Department of Medicine Haugesund Hospital Haugesund Norway; ^6^ Department of Pharmacy University of Oslo Oslo Norway

**Keywords:** kidney, PTDM, T2DM, tubuli

## Abstract

**Purpose:**

Diabetes mellitus (DM) is a major cause of end‐stage kidney disease (ESKD), with kidney transplantation being the preferred treatment. However, post‐transplant diabetes mellitus (PTDM) increases mortality and graft loss. While PTDM and Type 2 diabetes mellitus (T2DM) share risk factors, their mechanisms differ, particularly in diabetic nephropathy (DN). This study aimed to investigate the molecular differences in PTDM by mapping the proteomes of proximal tubuli and serum in normoglycemic (NG), pre‐transplant T2DM, and PTDM patients one year post‐transplantation. Experimental Design Proteomic analysis was performed on microdissected proximal tubular cells and serum samples from kidney transplant recipients categorized as NG, pre‐transplant T2DM, or PTDM at one year post‐transplantation. Mass spectrometry was used to identify differentially expressed proteins. Data analyses were performed using gene ontology databases and pathway analysis.

**Results:**

Proteomic analysis revealed key differences, including significant dysregulation of mitochondrial proteins and lipid metabolism pathways in PTDM patients compared to T2DM and NG groups. Additionally, we observed distinct serum patterns of cholesterol metabolism dysregulation in PTDM, highlighting a complex interplay between fatty acid metabolism, mitochondrial dysfunction and systemic lipid dysregulation that may drive renal injury in PTDM‐related DN.

**Conclusions and Clinical Relevance:**

This pilot study is the first to perform proteomic analysis on both microdissected tubular cells and serum from post‐transplant PTDM, pre‐transplant T2DM and NG transplant recipients. The proteomic differences between PTDM and T2DM could help to develop targeted therapies and early diagnostic markers, ultimately improving transplant outcomes and patient management. Further research is needed to validate these findings and explore their therapeutic potential.

**Abbreviations**
ACN: acetonitrile
aPC: activated Protein C
ApoA2: apolipoprotein A2
ApoB100: apolipoprotein B100
BMI: body mass index
CKD‐EPI: Chronic Kidney Disease Epidemiology Collaboration
DKD: diabetic kidney disease
DM: diabetes mellitus
DN: diabetic nephropathy
eGFR: estimated glomerular filtration rate
ESKD: end stage kidney disease
FA: formic acid
FFPE: formalin fixed and paraffin embedded
HbA1c: glycated haemoglobin
KEGG: Kyoto Encyclopedia of Genes and Genomes
LC: liquid chromatography
LDL: low density lipoproteins
LPL: apolipoprotein A
MS: mass spectrometry
NCE: normalized collision energy
NG: normoglycemic
OGTT: oral glucose tolerance test
PCA: principal component analysis
PROBE: Proteomics Unit at the University of Bergen
PTDM: post‐transplant diabetes mellitus
REC: Regional Ethics Committee
T2DM: Type 2 diabetes mellitus
TMT: tandem mass tags

## 1 Introduction

Diabetes mellitus (DM) is a leading cause of end‐stage kidney disease (ESKD) [1]. Treatment options for ESKD patients include dialysis and transplantation, with transplantation offering superior patient survival, quality of life and economic benefits [2].

**Summary**

• This study provides novel insights into the distinct molecular mechanisms underlying post‐transplant diabetes mellitus (PTDM) and pre‐existing Type 2 diabetes mellitus (T2DM) in kidney transplant recipients.
• By employing proteomic analysis of microdissected proximal tubuli and serum, we identified key differences in mitochondrial function and lipid metabolism pathways that may contribute to the early stages of diabetic nephropathy (DN).
• These findings highlight the potential for developing early diagnostic biomarkers and targeted therapies for PTDM, offering new avenues for improving long‐term transplant outcomes.
• This research enhances our understanding of the proteomic landscape in PTDM, paving the way for future studies focused on therapeutic intervention and personalized medicine in diabetic kidney disease.

Post‐transplant diabetes mellitus (PTDM) poses a significant challenge in the realm of solid organ transplantation, particularly in kidney transplantation. Approximately, 10%–20% of kidney transplant recipients develop PTDM, which is associated with increased mortality, infections, cardiovascular morbidity and reduced kidney graft survival [3, 4]. The risk factors for PTDM overlap with those for already pre‐existing Type 2 diabetes mellitus (T2DM) [3], and the glucose‐driven increase in glomerular filtration and tubular reabsorption may lead to an overload of the nephrons and kidney damage [5]. Consequently, both PTDM and T2DM may cause diabetic nephropathy (DN), with an incidence of 25% and 30%, respectively, within 6 years post‐transplantation [6].

Among patients with DM, 20%–40% develop DN, thus making it a common complication among both transplanted and non‐transplanted patients [7]. Indeed, DM is the leading cause of ESKD worldwide [8]. Improved prevention and treatment of patients with DM have led to a substantial reduction in diabetes‐related complications, such as DN and ESKD. However, despite advances in prevention and treatment that have reduced diabetes‐related complications, the increased prevalence of diabetes continues to impose a significant disease burden [9].

Although PTDM and T2DM share risk factors, their pathophysiology and clinical course differ [3]. DN is generally characterized by thickening of the glomerular basement membrane, mesangial matrix expansion, nodular glomerulosclerosis and arteriolar hyalinosis [5]. On the other hand, kidney biopsies from PTDM patients may also show allograft rejection‐induced tubulointerstitial and vascular alterations, as well as histological features associated with viral infections or immunosuppressive drug‐related toxicity [10]. Additionally, PTDM patients may experience diabetic complications, such as nephropathy, ketoacidosis and ophthalmic or neurological complications, earlier than non‐transplanted T2DM patients [11].

The differences in kidney biopsy findings and clinical course suggest that non‐identical mechanisms may be involved in the pathophysiology of recurrent kidney injury in recipients with T2DM pre‐transplantation and those developing PTDM. Thus, understanding their pathophysiological differences is crucial for early detection and effective therapy.

A way of investigating these differences is through proteomic studies. By studying the entire proteome, without relying solely on pre‐existing pathophysiologic knowledge, novel protein markers may be identified [12]. This is typically performed by using blood and/or urine [12, 13] due to their availability in the clinic and since liquid samples are easier to obtain than kidney tissue. However, investigating the proteome of tissue from kidney biopsies could provide a deeper understanding of specific tissue characteristics in normal and pathological states [14].

In this study, we compared the proteomes of proximal tubuli and serum from normoglycemic (NG), pre‐transplant T2DM and PTDM recipients 1 year after kidney transplantation with the aim to identify novel early biomarkers prior to the development of histologically visible DN. This approach, together with our previously published data of the proteomes of glomeruli from similar recipients’ groups [1, 15], seeks to enhance our understanding of the distinct pathophysiological mechanisms underlying recurrent kidney injury in PTDM and T2DM patients' post‐transplantation, ultimately aiding in the early detection and targeted treatment of these conditions.

## 2 Materials and Methods

### 2.1 Study Design and Patients

This study was approved by the Regional Ethics Committee (REC) of the South‐Eastern Norway Regional Health Authority (REC South‐East: 2016/912), and all patients gave written informed consent that the data and the samples can be used for research. All methods used in this manuscript were carried out in accordance with relevant guidelines and regulations.

Adult kidney transplant recipients were subjected to an in‐depth investigation 8–10 weeks and 1 year after transplantation, including oral glucose tolerance test (OGTT) and glycated haemoglobin (HbA1c) test, in addition to ultrasound‐guided renal biopsies, obtained using an 18‐gauge needle. Clinical data were stored in the transplant registry at Oslo University Hospital—Rikshospitalet, while the biopsies were stored as formalin‐fixed and paraffin‐embedded (FFPE) tissue in the Diagnostic and Treatment biobank‐Nyrefysiologisk laboratorium for conservation and further analyses.

We selected patients with a valid glucose metabolism status and a protocol biopsy at 1 year post‐transplantation, stable renal function with less than 20% deviation in serum creatinine within the last 2 months and stable immunosuppressive therapy for more than 3 months before protocol biopsy at 1 year examination. Immunosuppression was based on prednisolone, tacrolimus and mycophenolate mofetil, which is the standard immunosuppressive protocol in Norway [16], and was similar in all groups. Patients with PTDM and T2DM received insulin and other antidiabetics. Exclusion criteria were an estimated glomerular filtration rate (eGFR) < 30 mL/min/1.73 m^2^ (calculated using the Chronic Kidney Disease Epidemiology Collaboration [CKD‐EPI] formula), in addition to any clinical and/or histological manifestations of graft rejection during the first year after transplantation.

Three different patient groups were analysed: patients with PTDM (tubuli, *n* = 6; serum, *n* = 7), patients with pre‐transplant T2DM (tubuli, *n* = 6; serum, *n* = 8) and NG patients (tubuli, *n* = 5; serum, *n* = 8). Interstitial fibrosis and tubular atrophy in allograft biopsies were classified using Banff classification [17].

### 2.2 Laser Capture Microdissection and Proteomics Sample Preparation From FFPE Tissue

The kidney biopsy specimens were stored as FFPE tissue. Ten‐micrometer thick sections were deparaffinized, rehydrated, stained and scanned with ScanScope XT Aperio. Selected FFPE sections were mounted on pre‐irradiated polyethylene naphthalate slides (MembraneSlide 1.0 PEN, Carl Zeiss MicroImaging GmbH). Laser‐capture microdissection for isolation of a total area of approximately 2 × 10^6^ µm^2^ proximal tubuli was carried out using Leica LMD7 (Wetzlar, Germany). The tubuli were pressure catapulted into a tube cap (cat. no. 11600296, Leica, Wetzlar, Germany). Microdissected FFPE proximal tubuli were stored in 10 µL of RIPA lysis buffer (cat. no. R0278; Sigma‐Aldrich) with complete Protease Inhibitor Cocktail (cat. no. 04693116001; Roche) at −80°C until peptide extraction. The samples were then alkylated with 200 mM iodoacetamide (30 min at room temperature, dark) and digested overnight at 37°C with SP3 buffer (4 M urea, 100 mM HEPES, pH 8.5, in water) and a mass spectrometry grade Lys‐C and Trypsin mix with a concentration of 0.1 µg/µL (cat. no. V5071, Promega, Madison, WI, USA). Following digestion, samples were eluted with 2% DMSO, acidified with 1% methanoic acid and desalted in a 2 mg Oasis C18 96‐well plate (cat. no. 186001829, Waters, Milford, MA, USA). Samples were eluted with 80% acetonitrile, 0.1% methanoic acid and dried by a vacuum centrifuge before being resuspended in 0.5% formic acid and 2% acetonitrile. Next, the samples were tagged using tandem mass tag (TMT) dissolved in anhydrous acetonitrile (5 µg/µL). The samples were then combined, dried by a vacuum centrifuge and desalted in a HLB Oasis 96‐well plate (cat. no. 186000128, Waters, Milford, MA, USA). Subsequently, the combined samples were dried in a vacuum centrifuge. We determined the final peptide concentration using a NanoDrop Spectrophotometer (ThermoFisher).

### 2.3 Liquid Chromatography Tandem Mass Spectrometry Analysis From FFPE Tissue

Liquid Chromatography‐Tandem Mass Spectrometry (LC‐MS) analysis was performed as described in Aasebø et al. [18]. We dissolved 0.5 µg protein as tryptic peptides in 2% acetonitrile and 0.5% formic acid. Subsequently, the solution was injected in an Ultimate 3000 RSLC system (Thermo Scientific, Sunnyvale, CA, USA), connected online to a linear quadrupole ion trap‐orbitrap (LTQ‐Orbitrap Elite) mass spectrometer (Thermo Scientific, Bremen, Germany) equipped with a nanospray Flex ion source (Thermo Scientific, Sunnyvale, CA, USA). The samples were loaded and desalted on a pre‐column (Acclaim PepMap 100, 2 cm × 75 µm ID nanoViper column, packed with 3 µm C18 beads) at a flow rate of 6 µL/min for 5 min with 0.1% (vol/vol) trifluoroacetic acid.

### 2.4 Protein Extraction From Serum Samples

For protein extraction from serum samples, the Preomics ENRICH‐iST kit for mammalian blood plasma and serum was used, according to the manufacturer's protocol. Briefly, the serum samples were thoroughly mixed using paramagnetic beads that bind serum proteins, followed by enrichment facilitating the protein binding onto the beads. Lysis, reduction, alkylation, digestion and purification followed.

### 2.5 Proteomics Analysis From Serum Samples

Mass spectrometry‐based proteomic analyses were performed by the Proteomics Unit at the University of Bergen (PROBE). About 0.8 µg of tryptic peptides were dissolved in 2% acetonitrile (ACN) and 0.5% formic acid (FA) and injected into an Ultimate 3000 RSLC system (Thermo Scientific, Sunnyvale, CA, USA) connected online to a Exploris 480 mass spectrometer (Thermo Scientific, Bremen, Germany) equipped with EASY‐spray nano‐electrospray ion source (Thermo Scientific). The sample was loaded and desalted on a pre‐column (Acclaim PepMap 100, 2 cm × 75 µm ID nanoViper column, packed with 3 µm C18 beads) with 0.1% trifluoroacetic acid. Peptides were separated during a biphasic ACN gradient from two nanoflow UPLC pumps on a 25‐cm analytical column (PepMap RSLC, 25 cm × 75 µm ID. EASY‐spray column, packed with 2 µm C18 beads). Solvents A and B were 0.1% FA (vol/vol) in water and 100% ACN, respectively. The gradient composition was 5% B during trapping followed by 5%–8% B, 8%–22% B, 22%–32% B, and 32%–90% B. Elution of very hydrophobic peptides were performed with isocratic elution with 90% B. The analytical program was followed by the wash program with 6 µL 100% isopropanol, and then the trap column was conditioned by 5% B from the nanoflow pumps. Instrument control was through Thermo Scientific SII for Xcalibur 1.6. The FAIMS Pro interface performed gas‐phase fractionation, enabling the preferred accumulation of multiply charged ions to maximize the acquisition efficiency. Short‐ion residence time in the FAIMS Pro interface electrode assembly enables the use of multiple CV settings in a single run to increase proteome coverage. Peptides eluted from the column were detected in the Exploris 480 Mass Spectrometer. The mass spectrometer was operated in the DIA‐mode (data‐independent‐acquisition). Instrument control was through Orbitrap Eclipse Tune 3.5 and Xcalibur 4.5. Using an isolation window of 12 Da, all ions in the *m*/*z* window were sequentially isolated in the C‐trap before higher‐energy collision dissociation (HCD) fragmentation. Fragmentation was performed with a normalized collision energy (NCE) of 30%, and fragments were detected in the Orbitrap at a resolution of 15,000 at *m*/*z* 200. Lock‐mass internal calibration was not enabled.

### 2.6 Statistics and Computational Analysis

Processing of raw mass spectrometric data was performed using Proteome Discover Software (Thermo‐Fisher). Tandem mass spectra were searched against the human Universal Protein Resources (UniProt). Label‐free quantification was used to identify the number of proteins in each sample. Proteome analysis, PCA and hierarchical clustering were performed using Perseus (v. 1.5.5.3, RRID:SCR_015753). Briefly, data were filtered and transformed (log2 (*x*)). Rows with < 70% valid values in at least one group were excluded. Imputation of missing data was performed by random numbers drawn from a normal distribution with a width of 0.3 and downshift of 1.8 applied to each expression column separately, and data were normalized using *Z*‐score. The MS proteomics data have been deposited to the ProteomeXchange Consortium (http://proteomecentral.proteomexchange.org) [19] via the PRIDE partner repository with the dataset identifiers PXD054937 for microdissected tubuli and PXD054961 for serum samples. The mass spectrometry data were normalized before comparative statistical analysis. SPSS (IBM SPSS Statistics v.25; RRID:SCR_019096) was used for general statistics. The Welch's *t*‐test and ANOVA test were used for data comparisons, and *p* values ≤ 0.05 were considered statistically significant Given this biological variability and limited tissue availability, pathway‐level insights provide more robust conclusions. Our *p* value threshold (*p* < 0.05) was set to identify broader trends rather than specific protein changes, minimising the risk of false positives in this context.

ShinyGo was used for graphical gene ontology enrichment analysis, and Kyoto Encyclopedia of Genes and Genomes (KEGG) was used for the systematic analysis of gene functions.

## 3 Results

### 3.1 Sample Selection and Analysis

Proteomic analysis was performed on tubuli isolated from FFPE kidney biopsies and on serum sampled 1 year after kidney transplantation. The study involved three groups of adult patients: NG patients, patients with PTDM and patients with pre‐transplantation T2DM. Tables 1 and 2 summarize the clinical characteristics of the patients included in the final analysis for microdissected tubuli and serum, respectively. At the time of the biopsy, patients showed no signs of graft dysfunction, including any forms of rejection, micro‐ or macrovascular complications. T2DM patients were diagnosed with DM between 2 and 31 years prior to transplantation. For NG patients, a follow‐up conducted 4–5 years after biopsy showed that none of them had developed DM.

**TABLE 1:** Clinical characteristics of the final microdissected tubuli cohort.

	PTDM (*n* = 6)	T2DM (*n* = 6)	NG (*n* = 5)	*p* value PTDM vs. T2DM	*p* value PTDM vs. NG	*p* value T2DM vs. NG
Sex, male/female	4/2	5/1	4/1			
Age (years), mean (±SD)	64.5 (7.3)	65.3 (6.5)	64.2 (13.4)	0.852	0.966	0.871
Range	55–76	56–74	48–84			
BMI (kg/m^2^), mean (±SD)	27.8 (1.6)	30.5 (1.5)	26.1 (3.0)	0.020	0.357	0.039
eGFR (mL/min/1.73 m^2^), mean (±SD) post‐tx	52.0 (9.9)	48.5 (13.8)	43.2 (13.2)	0.656	0.304	0.573
*HBa1C (%)*, mean (±SD) post‐tx	6.66 (0.9)^*^	7.4 (1.0)^**^	5.4 (0.5)^***^	0.298	0.037	0.018

Abbreviations: BMI, body mass index; HbA1C, glycated haemoglobin; post‐tx, post‐treatment.

* *n* = 5.

** *n* = 5.

*** *n* = 3.

**TABLE 2:** Clinical characteristics of the final serum cohort.

	PTDM (*n* = 7)	T2DM (*n* = 8)	NG (*n* = 8)	*p* value PTDM vs. T2DM	*p* value PTDM vs. NG	*p* value T2DM vs. NG
Sex, male/female	4/3	5/3	5/3			
Age (years), mean (±SD)	64.0 (10.1)	65.8 (10.2)	55.75 (6.7)	0.761	0.118	0.050
Range	46–76	52–81	48–67			
BMI (kg/m^2^), mean (±SD)	27.1 (1.6)	30.1 (1.3)	25.8 (2.2)	0.003	0.218	< 0.001
eGFR (mL/min/1.73 m^2^), mean (±SD) post‐tx	51.9 (8.7)	44.3 (14.3)	53.8 (14.4)	0.262	0.776	0.118
*HBa1C* (%), mean (±SD) post‐tx	7.0 (0.7)	7.3 (0.8)	5.3 (0.3)	0.352	< 0.001	< 0.001

Abbreviations: BMI, body mass index; HbA1C, glycated haemoglobin; post‐tx, post‐treatment.

### 3.2 Tubular Proteomic Profiling and Identification of Differentially Abundant Proteins

**FIGURE 1:**
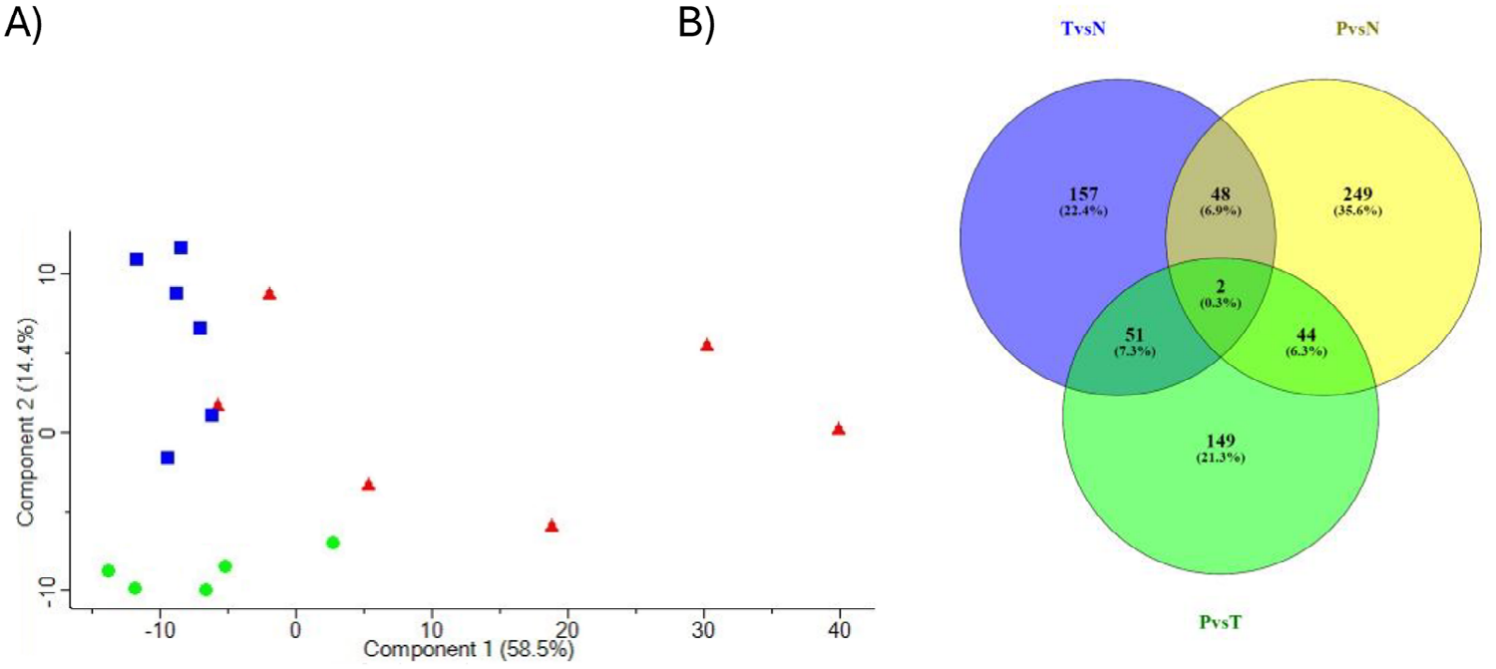
NG, PTDM, and T2DM tubuli protein analysis. (A) Protein principal component analysis (PCA) based on protein data from PTDM (blue dots), T2DM (red dots) and NG (green dots). (B) Venn diagram depicting the overlap of proteins differentially expressed in the three statistical comparisons obtained using http://bioinfogp.cnb.csic.es/tools/venny.

A collective count of 4359 proteins were identified in the tubular tissue samples, each distinguishable by at least one unique peptide sequence and thus selected further analyses. The identified proteins exhibited molecular masses ranging between 51 and 38,137 kDa. The proteome analysis showed that 343, 256 and 246 tubular proteins were differentially abundant in NG compared to PTDM, in NG compared to T2DM and in PTDM compared to T2DM, respectively. A two‐dimensional principal component analysis (PCA) was used to perform an initial exploratory assessment of the dataset based on ANOVA significantly differentially regulated proteins that indicated a clear separation of PTDM and NG groups along with principal component 2 (PC2); however, T2DM group presented a higher dispersion and some overlap with PTDM (Figure 1A). The proteins in common in each comparison are represented in Figure 1B. The data suggest that variations in the tubular proteomes may allow discrimination among these groups.

### 3.3 Differentially Abundant Proteins in T2DM and PTDM Tubuli

**FIGURE 2:**
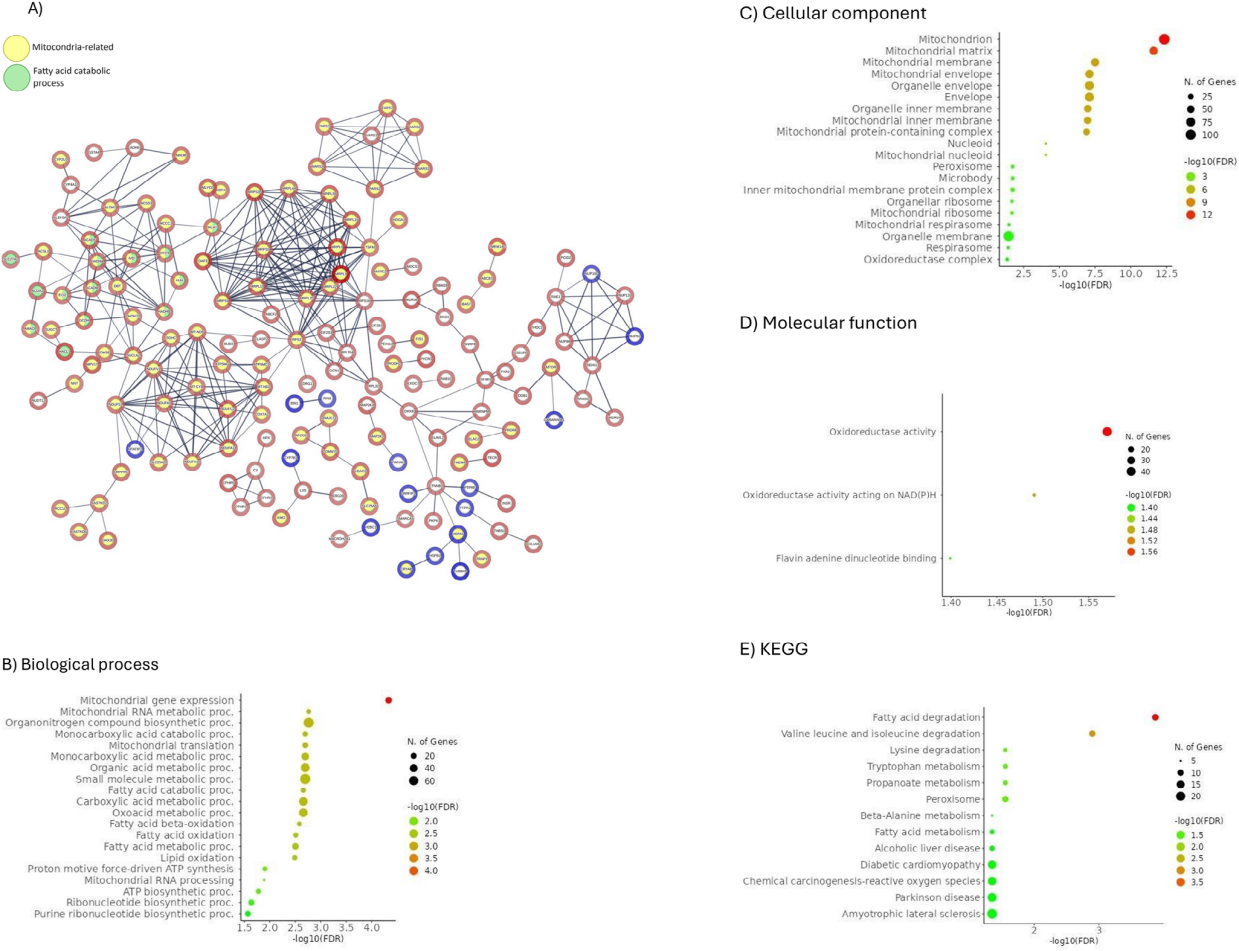
PTDM and T2DM pathway enrichment analyses. (A) Protein–protein interaction network using proteins with significantly different abundance in PTDM and T2DM. Only high‐confidence interactions were represented. Disconnected nodes are not shown. The network nodes represent specific proteins. Yellow inside the nodes represents mitochondria‐related proteins, whereas green is a fatty acid catabolic process. The red halo around the node refers to protein abundance higher in PTDM versus T2DM; blue halo refers to lower abundance in PTDM versus T2DM. Edges represent protein–protein associations. (http://www.string‐db.org). (B) GO biological process pathway enrichment analysis of proteins differentially expressed in PTDM versus T2DM. (C) Cellular component pathway enrichment analysis of proteins differentially expressed in PTDM versus T2DM. (D) Molecular function pathway enrichment analysis of proteins differentially expressed in PTDM versus T2DM. (E) KEGG pathway enrichment analysis of proteins differentially expressed in PTDM versus T2DM. The 20 most enriched pathways are represented (http://bioinformatics.sdstate.edu/go/).

To investigate potential differences in the pathways implicated in these two distinguishable conditions and detect potential PTDM and/or T2DM specific markers, we conducted category enrichment analysis on proteins showing a differential abundance in tubuli from T2DM and PTDM patients. In total, 256 tubular proteins were differentially abundant in T2DM compared to PTDM. From them, 207 proteins were found to be more abundant in tubuli from PTDM patients compared to those from T2DM patients. We found that proteins related to mitochondrial gene expression, mitochondrial RNA metabolic process, mitochondrial RNA processing and fatty acid degradation, between others, were significantly differently expressed in tubuli from PTDM patients compared to T2DM patients, being in general higher in PTDM (Figure 2A–E). In general, mitochondrial ribosomal proteins (e.g., MRPL3, MRPL15, MRPL9, MRPS35 and DAP3) were more abundant in PTDM in comparison to T2DM.

### 3.4 Differentially Abundant Proteins in NG and PTDM Tubuli

**FIGURE 3:**
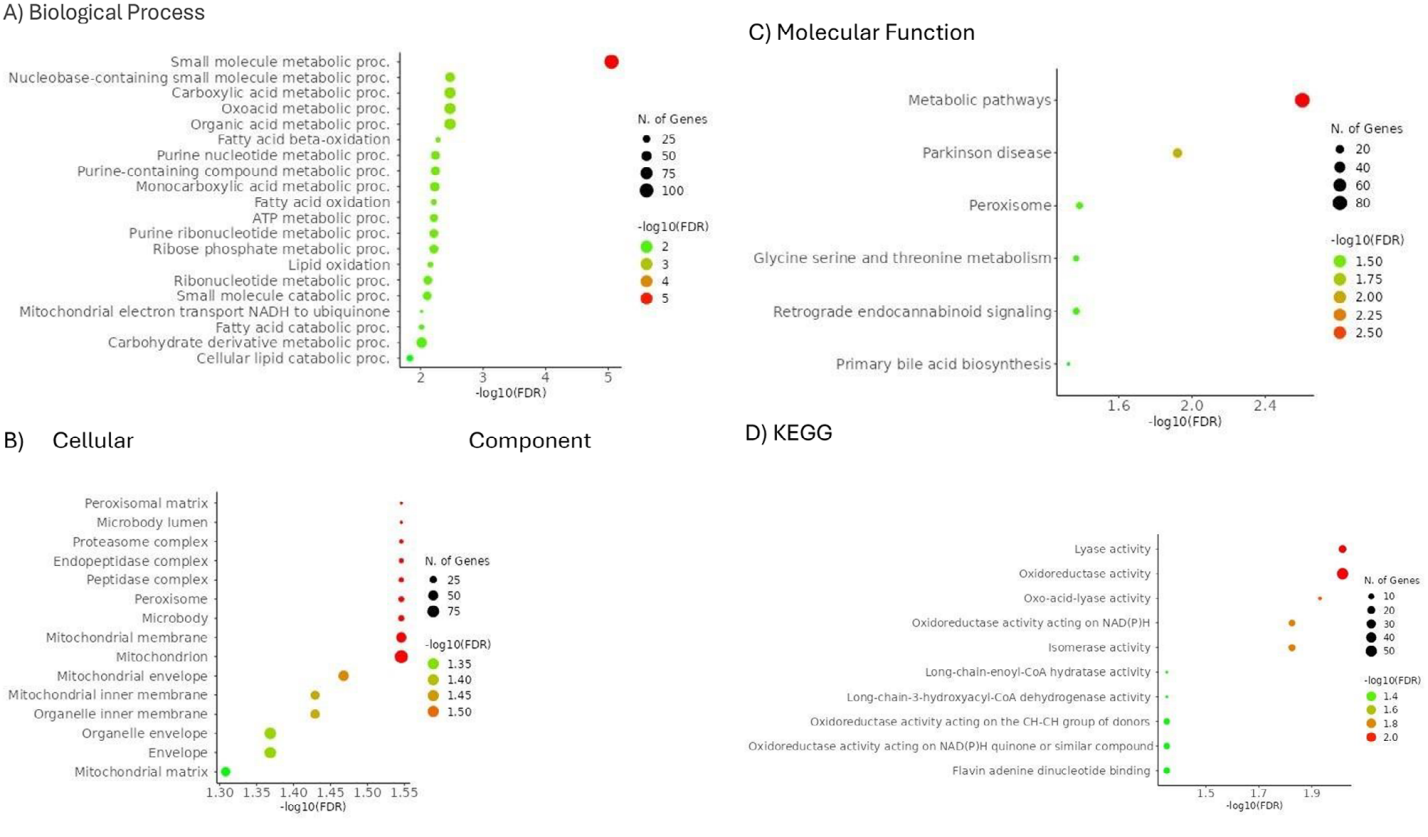
PTDM and NG pathway enrichment analyses in tubuli. (A) GO biological process pathway enrichment analysis of proteins differentially expressed in PTDM versus NG. (B) Cellular component pathway enrichment analysis of proteins differentially expressed in PTDM versus NG. (C) Molecular function pathway enrichment analysis of proteins differentially expressed in PTDM versus NG. (D) KEGG pathway enrichment analysis of proteins differentially expressed in PTDM versus NG. The 20 most enriched pathways are represented (http://bioinformatics.sdstate.edu/go/).

Specific effects related to kidney transplantation, including the use of immunosuppressive treatments, may contribute to the development of PTDM. To gain understanding of potential pathways implicated, we conducted category enrichment analysis on proteins showing differential abundance in tubuli from NG and PTDM patients. In total, 343 tubular proteins were differentially abundant in NG compared to PTDM. From them, 159 proteins were found to be more abundant in tubuli from NG compared to those from PTDM patients. The disrupted pathways included small molecule metabolic processes, lyase and oxidoreductase activities (Figure 3A–D). Interestingly, the mitochondrial ribosomal proteins were also higher in PTDM than NG.

### 3.5 Serum Proteomic Profiling and Identification of Differentially Abundant Proteins

**FIGURE 4:**
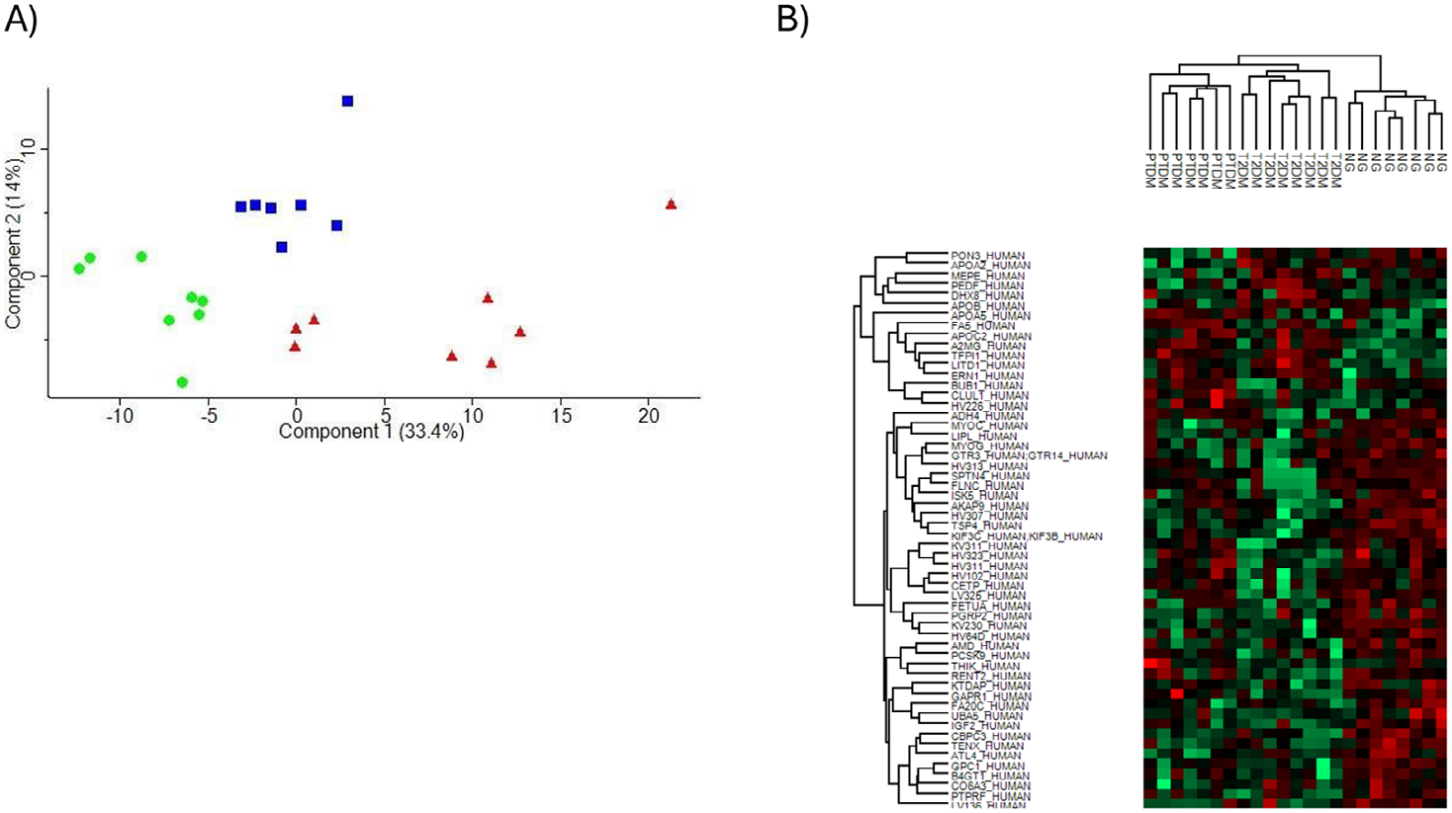
NG, PTDM, and T2DM serum protein analysis. (A) Protein principal component analysis (PCA) based on protein data from PTDM (blue dots), T2DM (red dots) and NG (green dots). (B) Hierarchical clustering of proteins differentially expressed in serum from patients with PTDM, T2DM and NG.

A collective count of 678 proteins was distinguishable by at least one unique peptide sequence and thus selected further analyses. The data suggest that variations in the serum proteomes may allow discrimination among these three groups. An initial proteome analysis showed that 35, 62 and 30 serum proteins were differentially abundant in NG compared to PTDM, in NG compared to T2DM and in PTDM compared to T2DM, respectively. Of them, 11 out of 62 proteins differentially abundant In NG compared to T2DM were not mapped, and 3 out of 62 corresponded to immunoglobulins that were not included in the ontology analysis. A two‐dimensional PCA was used to perform initial exploratory assessment of the dataset based on ANOVA significantly differentially regulated proteins. A clear separation between the three groups under investigation was evident when the samples were plotted on these two axes (Figure 4A). For the identification of specific expression patterns in the serum proteomic analysis, hierarchical clustering of proteins differentially abundant in the PTDM, T2DM and NG groups was performed (Figure 4B).

### 3.6 Differentially Abundant Proteins in T2DM and PTDM Serum

**FIGURE 5:**
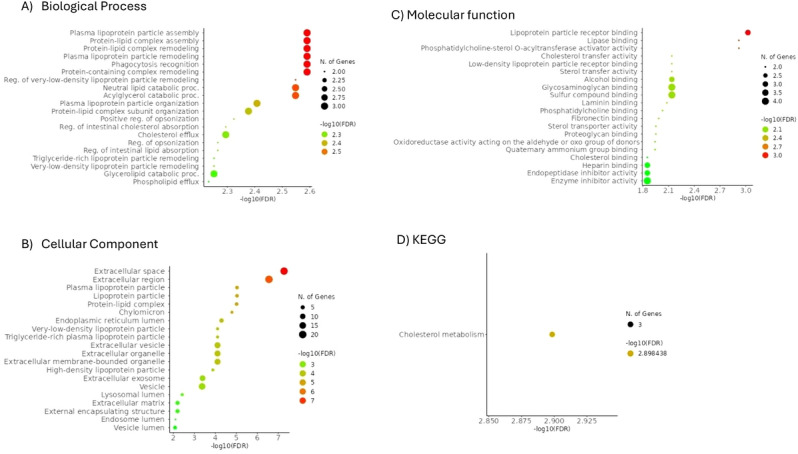
PTDM versus T2MD pathway enrichment analyses in serum. (A) GO biological process pathway enrichment analysis of proteins differentially expressed in PTDM versus T2DM. (B) Cellular component pathway enrichment analysis of proteins differentially expressed in PTDM versus T2DM. (C) Molecular function pathway enrichment analysis of proteins differentially expressed in PTDM versus T2DM. (D) KEGG pathway enrichment analysis of proteins differentially expressed in PTDM versus T2DM. The 20 most enriched pathways are represented (http://bioinformatics.sdstate.edu/go/).

We conducted category enrichment analysis on proteins showing differential abundance in tubuli from T2DM and PTDM patients. In total, 31 proteins were differentially abundant in T2DM compared to PTDM. Figure 5A–D represents the pathway enrichment analysis for these proteins. Interestingly, the most significant pathways are specifically associated with proteins that were downregulated in serum from PTDM patients compared to T2DM patients, including cholesterol metabolism, vitamin digestion and absorption, fat digestion and absorption, PPAR signalling pathway and ECM–receptor interaction.

PTDM is characterized by `de novo’ development following transplantation. Although our data consistently documented commonalities between PTDM and T2DM serum proteomic profiles, we addressed the identification of the few differentially expressed proteins, potentially suggesting specificities of PTDM development. Indeed, coagulation factor V, apoA5 upregulated in PTDM compared to T2DM serum. In contrast, proteins that were downregulated in the serum of PTDM patients compared to the serum of T2DM patients included Apolipoprotein A2 (ApoA2) that may stabilize high‐density lipoprotein (HDL), Apolipoprotein B 100 (ApoB100), that is, a major protein constituent of chylomicrons, LDL, VLDL and Lipoprotein A (LPA).

### 3.7 Differentially Abundant Proteins in NG and PTDM Serum

**FIGURE 6:**
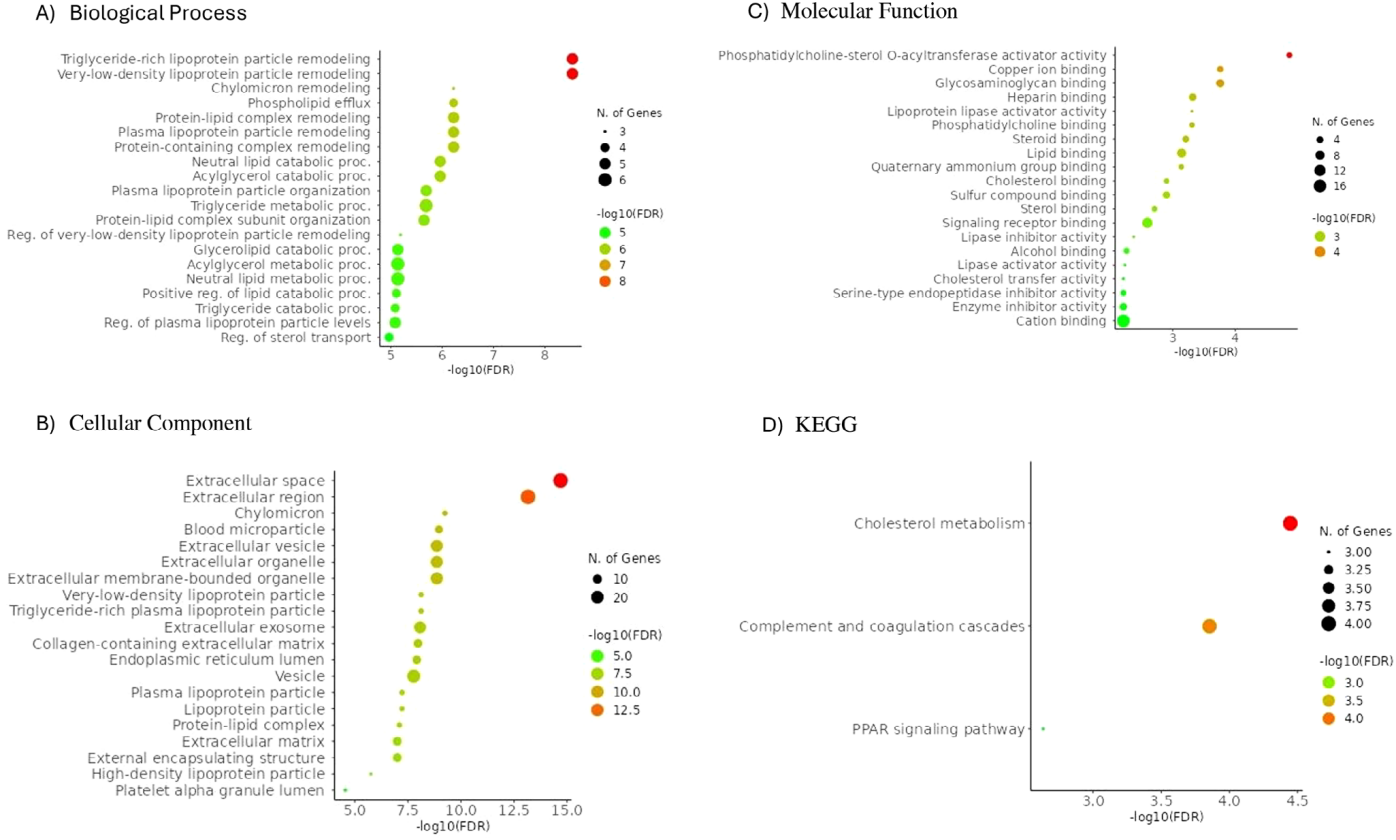
PTDM versus NG pathway enrichment analyses in serum. (A) GO biological process pathway enrichment analysis of proteins differentially expressed in PTDM versus NG. (C) Cellular component pathway enrichment analysis of proteins differentially expressed in PTDM versus NG. (D) Molecular function pathway enrichment analysis of proteins differentially expressed in PTDM versus NG. (E) KEGG pathway enrichment analysis of proteins differentially expressed in PTDM versus NG. The 20 most enriched pathways are represented (http://bioinformatics.sdstate.edu/go/).

We conducted category enrichment analysis on proteins showing differential abundance in serum from NG and PTDM patients. In total, 44 proteins were differentially abundant in NG compared to PTDM. Most proteins with differential abundance were found to be less abundant in serum from PTDM patients compared to this from NG patients. In Figure 6A–C, the general pathway analysis for these proteins is shown. Among others, proteins related to cholesterol metabolism, PPAR signalling pathway and lipoprotein particle remodelling were downregulated in serum from PTDM patients compared to NG patients. Proteins related to complement and coagulation cascades were upregulated in serum from PTDM patients compared to NG patients.

### 3.8 Nondiabetic and Diabetic Serum Proteomes in Kidney Transplant Patients: Gene Ontology and Protein Interaction Analysis

**FIGURE 7:**
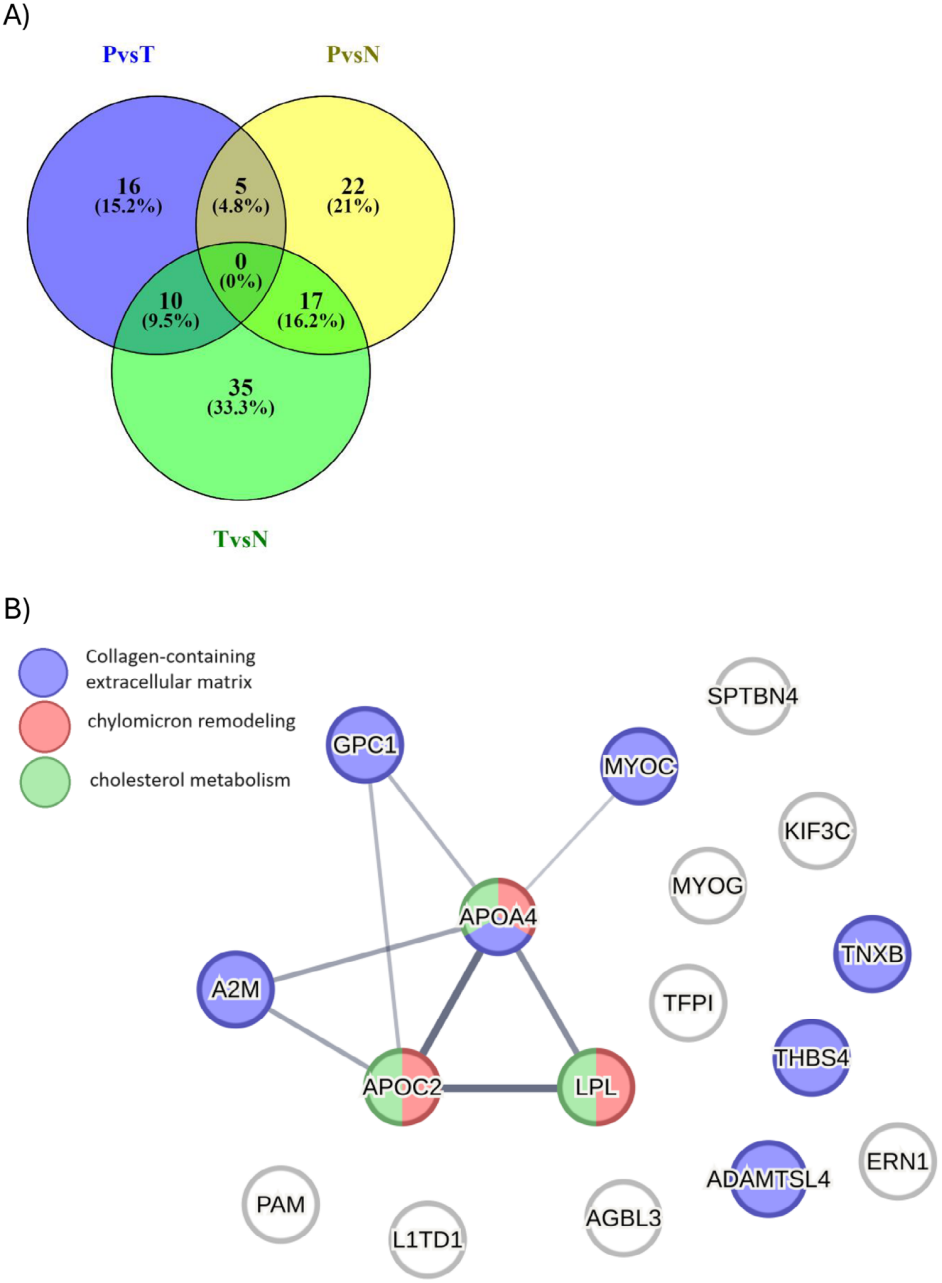
PTDM and T2DM versus NG serum protein analysis. (A) Venn diagram depicting the overlap of proteins differentially expressed in the three statistical comparisons obtained using http://bioinfogp.cnb.csic.es/tools/venny. (B) Protein–protein interaction network using proteins with significantly different abundance in PTDM and T2DM versus NG. The network nodes represent specific proteins. Red inside the nodes represents chylomicron remodelling‐related proteins, green is cholesterol metabolism and blue is the collagen‐containing extracellular matrix. Edges represent protein–protein associations. (http://www.string‐db.org).

Our study enabled proteomic profiling of serum from transplanted patients with DM, regardless of whether it preexisted or developed `de novo’ after the transplantation. Thus, we sought to identify proteins differentially abundant between NG glomeruli and the combination of PTDM and T2DM serum (Figure 7A). A category enrichment analysis was performed and revealed that, overall, these proteins were biologically connected as a group (Figure 7B). Several proteins were overrepresented in serum from NG patients compared to all DM patients. Notably, proteins included in the pathway of the cholesterol metabolism were more abundant in NG samples than in all DM samples, irrespectively of their PTDM and T2DM nature. In contrast, in the group of proteins less abundant in NG, no significant PPI enrichment was observed. Based on their significantly different expression between the PTDM and T2DM patients, apolipoprotein A (ApoA), ApoB48 and Apo100 could be potential markers to differentiate T2DM from PTDM in the future.

## 4 Discussion

This study provides a comprehensive proteomic analysis of kidney tubuli and serum from post‐transplant patients with PTDM, T2DM and NG. The findings highlight key molecular differences between PTDM and T2DM in both kidney tissue and blood. In PTDM tubuli, mitochondrial‐related proteins involved in energy metabolism and fatty acid degradation were more abundant compared to T2DM, suggesting mitochondrial dysfunction in PTDM. Meanwhile, serum proteomics revealed downregulation of cholesterol metabolism and lipid transport proteins in PTDM compared to T2DM, including ApoB100 and ApoA2. These findings point to distinct metabolic pathways in PTDM that affect both kidney function and systemic lipid regulation, linking mitochondrial dysfunction in kidney tubuli with lipid dysregulation in the blood.

The kidneys, particularly the proximal tubuli, require substantial amounts of energy to perform their functions. In fact, fatty acid beta‐oxidation in mitochondria is a critical pathway for energy production in renal cells. In our data, the dysregulation of enzymes involved in fatty acid degradation pathway suggests disruptions in cellular energy metabolism and lipid homeostasis in tubular cells of PTDM patients compared to those of T2DM and NG recipients. These disruptions suggest impaired cellular energy metabolism and lipid homeostasis, potentially contributing to renal injury and fibrosis in PTDM‐related DKD [20].

Furthermore, we also found that apolipoproteins, essential for lipid transport and metabolism, presented differential expression between PTDM and pre‐transplant T2DM kidney recipients. Specifically, ApoB48, was downregulated in PTDM compared to T2DM, highlighting its role in intestinal lipid transport in PTDM [21, 22]. The upregulation of ApoB100 in T2DM indicates potential disturbances in hepatic lipid transport and LDL synthesis. Also, ApoA, the main component of HDL which plays a key role in reverse cholesterol transport and protection against LDL oxidation has been implicated in glucagon secretion modulation [23], was upregulated in T2DM compared to PTDM. Interestingly, a pilot study has previously found that ApoA was upregulated in the glomeruli of patients with T2DM compared to PTDM, even though the study's focus was not on cholesterol metabolism. These findings aligned with our study's serum analysis and the suggested disrupted cholesterol metabolism in PTDM patients [15].

The difference in the mean body mass index (BMI) values between PTDM and T2DM patients should also be considered. Based on the World Health Organization (WHO) definition of BMI, the T2DM patients had a BMI in the range of obesity (BMI ≥ 30), whereas the PTDM and NG patients showed pre‐obesity (BMI ≥ 25 and < 30) (Table 1) [24]. Obesity is linked to cholesterol metabolism dysregulation [25], and this can be a contributing factor to the T2DM cholesterol metabolism downregulation. However, the proteomics analysis revealed cholesterol metabolism downregulation in the PTDM patients compared to the NG patients, even though the difference in the mean BMI values between PTDM and NG patients was not significant. Based on results retrieved from a systematic review on proteomics in obesity [26], 41 proteins have been identified to be related to obesity from proteomics analyses of various samples, including blood. Out of these 41 proteins, 7 were detected in our serum proteins data. Out of these, only ApoB100 displayed a significantly different expression between the three groups of patients. Although apoB100, a protein previously found to be related to obesity, had a significantly higher expression in T2DM compared to both PTDM (*p* = 0.015) and NG groups, the rest of the proteins that displayed a significantly different expression between T2DM and PTDM do not appear to be related to obesity. Therefore, we believe that the overall observed differences in protein expression are not confounded by BMI. These findings suggest that potentially the dysregulation of fat digestion and absorption is higher in T2DM compared to PTDM; and thus, there is a higher risk of atherosclerosis in these patients.

An increased production rate of chylomicrons contributes to the dyslipidaemia observed in insulin resistance and T2DM and potentially elevates the atherosclerosis risk [27]. The differential expression of apolipoproteins between PTDM and T2DM addresses the possibility of distinct lipid metabolic pathways involved in these conditions and underscores the importance of tailored therapeutic strategies. Additionally, targeted interventions aiming at normalising apolipoprotein levels and restoring lipid homeostasis may offer promising avenues for mitigating disease progression and improving patient outcomes in these populations [28, 29].

A notable finding of our study is the profound dysregulation of proteins associated with mitochondrial function and metabolism in tubular cells of PTDM patients compared to T2DM and NG patients. Mitochondria play a central role in energy production, oxidative metabolism and cellular homeostasis. The proximal tubular segment of the nephron uses substantial amounts of energy primarily in the form of adenosine triphosphate (ATP), generated in the mitochondria [30]. The dysregulated expression of mitochondrial ribosomal proteins, including MRPL3, MRPL15, MRPL9, MRPS35 and DAP3, suggests disruptions in mitochondrial protein synthesis, function and apoptosis regulation, implicating mitochondrial dysfunction in mediating renal injury and cell death in PTDM‐related DKD [31].

Thus, both the cholesterol metabolism and the mitochondrial function were found to be dysregulated in the tubuli from PTDM patients in our study. In addition to its ability to efflux cholesterol, HDL is believed to have anti‐inflammatory and antioxidant properties and decreases the formation of atheroma [32, 33]. Elevated levels of plasma cholesterol and triglycerides have been linked to the onset of mitochondrial dysfunction [34]. An inverse correlation between circulating levels of HDL and mitochondrial DNA damage implies that HDL contributes to maintaining mitochondrial integrity [35]. The cholesterol metabolism was also found to be dysregulated in PTDM patients in serum analysis.

In general, the proteomic analyses of both tubuli and serum in PTDM patients indicate a complex interplay between fatty acid metabolism, mitochondrial dysfunction and cholesterol metabolism. The higher levels of mitochondrial‐related proteins in PTDM tubuli can indicate an adaptive response to mitochondrial dysfunction, while the dysregulation of fatty acid metabolism enzymes suggests impaired energy production. Systemic lipid dysregulation, as evidenced by lower levels of cholesterol metabolism‐related proteins in serum, can increase renal lipotoxicity and further impair mitochondrial function.

This pilot study, to our knowledge, is the first to conduct proteomics analysis on both microdissected tubular cells and serum from post‐transplant PTDM, pre‐transplant T2DM and NG kidney transplant recipients. By revealing distinct proteomic profiles in the tubuli and serum of these patient groups, our findings enhance the understanding of early PTDM effects on kidney tubuli before histologically visible DKD onset. This facilitates the identification of new blood biomarkers differentiating PTDM from T2DM, ultimately aiming to develop more effective, patient‐specific treatments.

Several limitations of our study should be acknowledged. Firstly, the relatively small sample size in each group limits the statistical power of our analyses, increasing the risk of confounding factors, such as variations in medication and clinical parameters, influencing the results. While there were no significant differences in age or glomerular filtration rate (eGFR) between PTDM, T2DM and NG groups, the slightly younger cohort in the NG group and the higher BMI in T2DM patients could have influenced some of the proteomic profiles observed. Secondly, all patients were treated with tacrolimus, a drug known to increase the risk of diabetes mellitus post‐transplantation [36], and corticosteroids that are associated with elevated blood glucose levels and insulin resistance [37]. However, tacrolimus is widely used in kidney transplant recipients [38], making the impact of these treatments relevant to the clinical context. Finally, the lack of pre‐transplant and immediate post‐transplant samples limits our ability to assess the temporal changes in proteomic profiles over the course of transplantation and immunosuppressive therapy.

Blood and/or urine samples have been mainly utilized in proteomic studies in DKD [13]. However, post‐transplant tissue biopsies can provide valuable insights into specific tissue characteristics in normal and pathological conditions [14]. By revealing distinct proteomic profiles in the tubuli and serum of patients with PTDM, T2DM and NG, we enhance our understanding of the early effects of PTDM on the kidney tubuli before the onset of histologically visible DKD, and it also facilitates the identification of new blood biomarkers that differentiate PTDM from T2DM, ultimately aiming to develop more effective, patient‐specific treatments.

While patient heterogeneity and the limited statistical power must be considered, our study demonstrates that proteome quantitation can effectively differentiate between the three post‐transplantation groups. These preliminary results provide a foundation for further investigation into the suggested interplay between fatty acid metabolism, mitochondrial dysfunction, cholesterol metabolism and the pathogenesis of glomerular filtration barrier alterations in transplantation and DM development.

Key findings include mitochondrial dysfunction and dysregulated fatty acid metabolism in the tubuli of PTDM patients, alongside alterations in cholesterol and lipoprotein metabolism in the serum. These proteomic differences suggest an early impact of PTDM on kidney function before the onset of histologically detectable damage. Additionally, the identification of differential expression patterns in apolipoproteins and coagulation factors in serum highlights potential biomarkers distinguishing PTDM from T2DM. These results could be useful for more tailored, patient‐specific therapeutic approaches to mitigate kidney injury in post‐transplant diabetes. Ultimately, these findings may guide the development of more personalized treatments to improve outcomes for patients with post‐transplant diabetes.

### 4.1 Associated Data

The mass spectrometry proteomics data have been deposited to the ProteomeXchange Consortium [19] via the PRIDE [39] partner repository with the dataset identifiers PXD054937 for microdissecte tubuli and PXD054961 for serum samples. The clinical data that support the findings of this study are available on request from the corresponding author. Individual, deidentified participant data are not freely available because of the risk of patient deidentified. However, interested parties can request access to de‐deidentified participant data or anonymized clinical study reports through submission of a request for access to the corresponding author, provided that the necessary data protection agency and ethical committee approvals are provided in compliance with relevant legislation.

## Conflicts of Interest

The authors declare no conflicts of interest.

## Supporting information



Supporting Information

## Data Availability

The mass spectrometry proteomics data have been deposited to the ProteomeXchange Consortium via the PRIDE partner repository with the dataset identifiers PXD054937 for microdissecte tubuli and PXD054961 for serum samples. The clinical data that support the findings of this study are available on request from the corresponding author.
