# Supplementary material for: Proteome of Renal Tubuli and Serum Differentiate Pre‐Existing Type 2 Diabetes and Post‐Transplant Diabetes in Kidney Transplant Recipients
Source: Proteomics Clin Appl. 2025 Feb 24;19(3):e70000. doi: 10.1002/prca.70000 (PMC12069002; doi:10.1002/prca.70000)
Supplement: Supplementary file 1 — Supporting Information [file PRCA-19-e70000-s001.docx]

A)


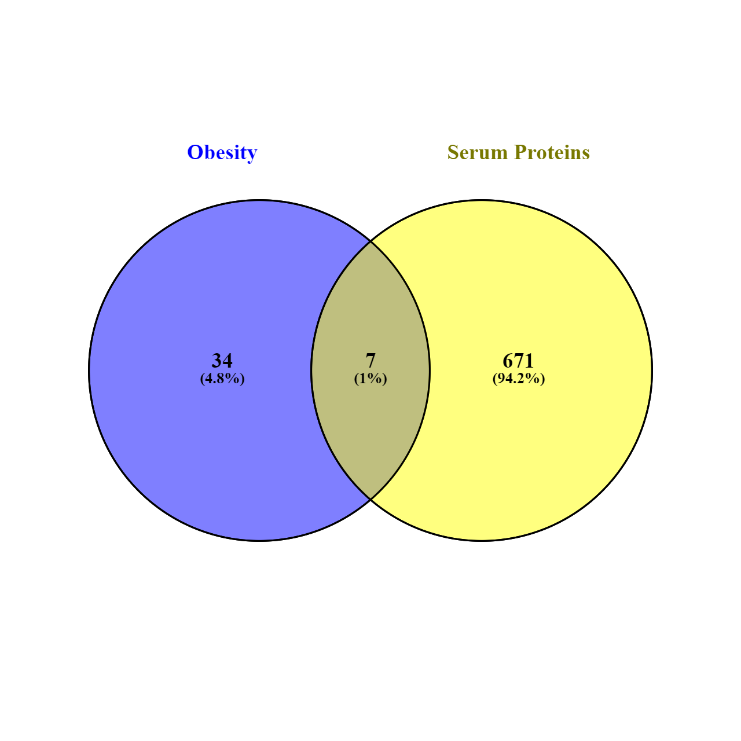


B)


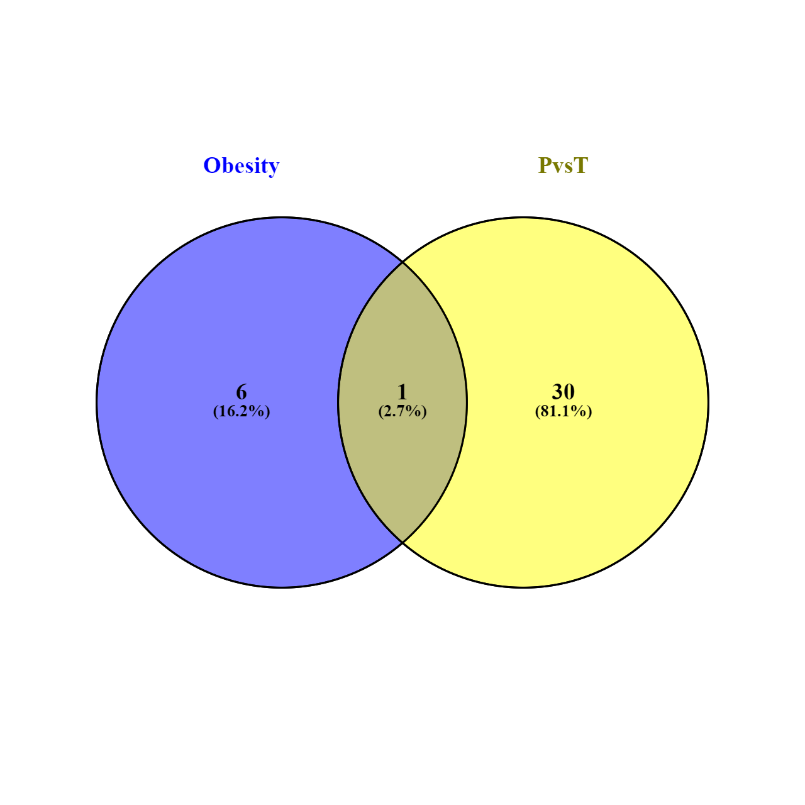


**Supplementary material.** Proteins related to Obesity. **A** Venn diagram depicting the overlap of proteins related to obesity and the proteins detected in serum. **B** Venn diagram depicting the overlap of proteins related to obesity and the significantly expressed proteins in common among PTDM and T2DM obtained using <http://bioinfogp.cnb.csic.es/tools/venny>.
